# An autopsy case of myocardial infarction due to idiopathic thrombotic thrombocytopenic purpura

**DOI:** 10.1186/s13000-015-0285-1

**Published:** 2015-05-29

**Authors:** Takashi Tasaki, Sohsuke Yamada, Atsunori Nabeshima, Hirotsugu Noguchi, Aya Nawata, Masanori Hisaoka, Yasuyuki Sasaguri, Toshiyuki Nakayama

**Affiliations:** Department of Pathology and Cell Biology, School of Medicine, University of Occupational and Environmental Health, 1-1 Iseigaoka, Yahatanishi-ku, Kitakyushu, 807-8555 Japan; Pathology and Oncology, University of Occupational and Environmental Health, Kitakyusyu, Japan; Department of Pathology, Fukuoka Wajiro Hospital, Fukuoka, Japan

**Keywords:** Pathology, Autopsy, Heart conduction system, Myocardial infarction, Thrombotic thrombocytopenic purpura, Arrhythmia

## Abstract

**Abstract:**

Thrombotic thrombocytopenic purpura (TTP) is a life-threatening disorder characterized by systemic platelet-von Willebrand factor aggregation, organ ischemia and profound thrombocytopenia. In this report, we describe an autopsy case of a 77-year-old Japanese man diagnosed with idiopathic TTP. He had no history of cardiovascular disease symptoms, such as chest pain, ST segment elevation, and elevation of cardiac enzyme levels, except arrhythmia. The patient suddenly died despite receiving many treatments. On autopsy, macroscopically and microscopically, acute and chronic myocardial infarction manifested as petechiae and fibrotic foci and covered a wide area in the myocardium, including the area near the atrioventricular node. The microthrombi in the small arterioles and capillaries were platelet thrombi, which showed positive results for periodic acid-Schiff stain and factor VIII on immunohistochemical staining. The cause of the sudden death was suspected to be myocardial infarction, including a cardiac conduction system disorder due to multiple platelet microthrombi. Asymptomatic myocardial infarction is an important cause of death in TTP. Therefore, the heart tissue, including the sinus-atrial node and the atrioventricular node, should be microscopically examined more closely in autopsy cases of patients with TTP who experienced sudden death of TTP. This report is a critical teaching case considering that its cause of sudden death may be arrhythmia due to a myocardial infarction including cardiac conduction system disorder by platelet microthrombi.

**Virtual Slides:**

The virtual slide(s) for this article can be found here: http://www.diagnosticpathology.diagnomx.eu/vs/2113354005156739

## Background

Thrombotic thrombocytopenic purpura (TTP) was first described by Moschcowitz in 1924 [1]. TTP is a rare disease with a reported incidence of 6 cases per million per year [2]. TTP patients are typically characterized by a pentad of symptom including microangiopathic hemolytic anemia, thrombocytopenia, neurological abnormalities, renal failure and pyrexia [3]. However, TTP patients rarely exhibit the full pentad. Idiopathic TTP is an autoimmune disease caused by severe deficiency of the von Willebrand factor (VWF) cleaving protease, a disintegrin-like and metalloprotease with thrombospondin type 1 repeats (ADAMTS13), due to inhibitory anti-ADAMTS13 antibodies [4,5]. ADAMTS13 prevents the normal processing of large VWF multimers that are secreted from endothelial cells [6], which is due to the microthrombi from massive platelet aggregation rather than the plaque rupture-thrombosis cascade [7]. Various causes of TTP have been identified (i.e., tumor, bacterial infection, human immunodeficiency virus [HIV] infection, collagen disease, and bone marrow transplantation); however, many cases of TTP are idiopathic.

TTP is often accompanied by microvascular ischemia and myocardial injury, which are observed in many patients with TTP. Pathological studies could be used to demonstrate cardiac involvement in TTP in >40% of cases [8-11]. Extensive myocardial microthrombi in patients who died of acute TTP were also observed in another postmortem study [8]. However, myocardial infarction with idiopathic TTP was observed to have no symptoms of angina or myocardial infarction, electrocardiograph (ECG) abnormalities (i.e., ST segment elevation), and elevated cardiac enzymes [7,8,12]. Angiograms for acute myocardial infarction and TTP have been described in the literature, but the majority of reported cases did not involve any significant pericardial diseases [13]. Clinically, it is very difficult to diagnose a myocardial infarction in TTP patients. Involvement of the heart in TTP patients sometimes can manifest as arrhythmias [14]. The incidence of arrhythmias (i.e. atrial fibrillation, any supraventricular tachyarrhythmias) has been reported to be higher in patients with myocardial infarction than in those without it [15]. However, the histopathological examination about arrhythmia of the TTP patient is not accomplished enough. In a previous report, the cardiac conduction system was involved in 70% of TTP cases according to cardiac autopsies, and relevant cardiac conduction system disorders only occurred in 20% of these patients [16].

In this report, we describe an autopsy case of idiopathic TTP with cardiac involvement, clinical atrial fibrillation, and microscopic myocardial multiple infarction without any evidence of coronary stenosis.

## Materials and methods

The patient was a 77-year-old Japanese man. The autopsy specimens after fixation in 10% neutral buffered formalin were embedded in paraffin for histological or immunohistochemical examinations. All immunohistochemical stainings were carried out using Dako Envision kit (Dako Cytomation Co., Glostrup, Denmark) according to the manufacturer’s instructions [17].

All histological and immunohistochemical slides were evaluated by two independent observers (certified surgical pathologists in our department) [17,18].

## Case presentation

### Clinical summary

The patient admitted to the hospital due to a cerebral lacuna infarction. He had no neurological sequelae. There was no clinical history of treatment with antiplatelet drugs ticlopidine and clopidogrel. The red blood cell and platelet count had been within normal limits. Ten months after the development of cerebral infarction, he suffered from a sudden headache, impaired consciousness, and severe fever (>39°C). Laboratory findings indicated thrombocytopenia (blood platelet count, 9000/μL) and hemolytic anemia (hemoglobin, 8.3 mg/dL; total-bilirubin 2.2 μL/mL; aspartate aminotransferase, 53 IU/mL; lactase dehydrogenase, 989 IU/mL), with red blood cell fragmentation on a peripheral blood smear. The inflammation reactions were elevated (white blood cell count, 7100/μL; C-reactive protein 3.0 mg/dL). Blood clotting factors and serum creatine levels were within normal limits. The direct and indirect Coombs tests yielded negative results. The test for the VWF cleaving protease inhibitor indicated a positive result and the VWF cleaving protease activity was undetectable. Important laboratory findings are listed in Table 1. He was clinically diagnosed with acute idiopathic TTP. He had no previous history of collagen disease, bacterial infection, HIV infection, or bone marrow transplantation. The patient was treated with plasma exchange therapy, steroid pulse therapy (methylprednisolone, 1000 mg/day), and rituximab (500 mg/day). However, his symptoms and physical state worsened rapidly. One day after initiating treatment, atrial fibrillation was monitored on an ECG. Cardiac manifestations, as included by chest pain and ST segment elevation, were not observed throughout the course of his clinical history. An echocardiography had not been performed. He died due to bradycardia and blood pressure reductions. An autopsy was performed 2 hours after his death.

### Pathological findings

**Figure 1:**
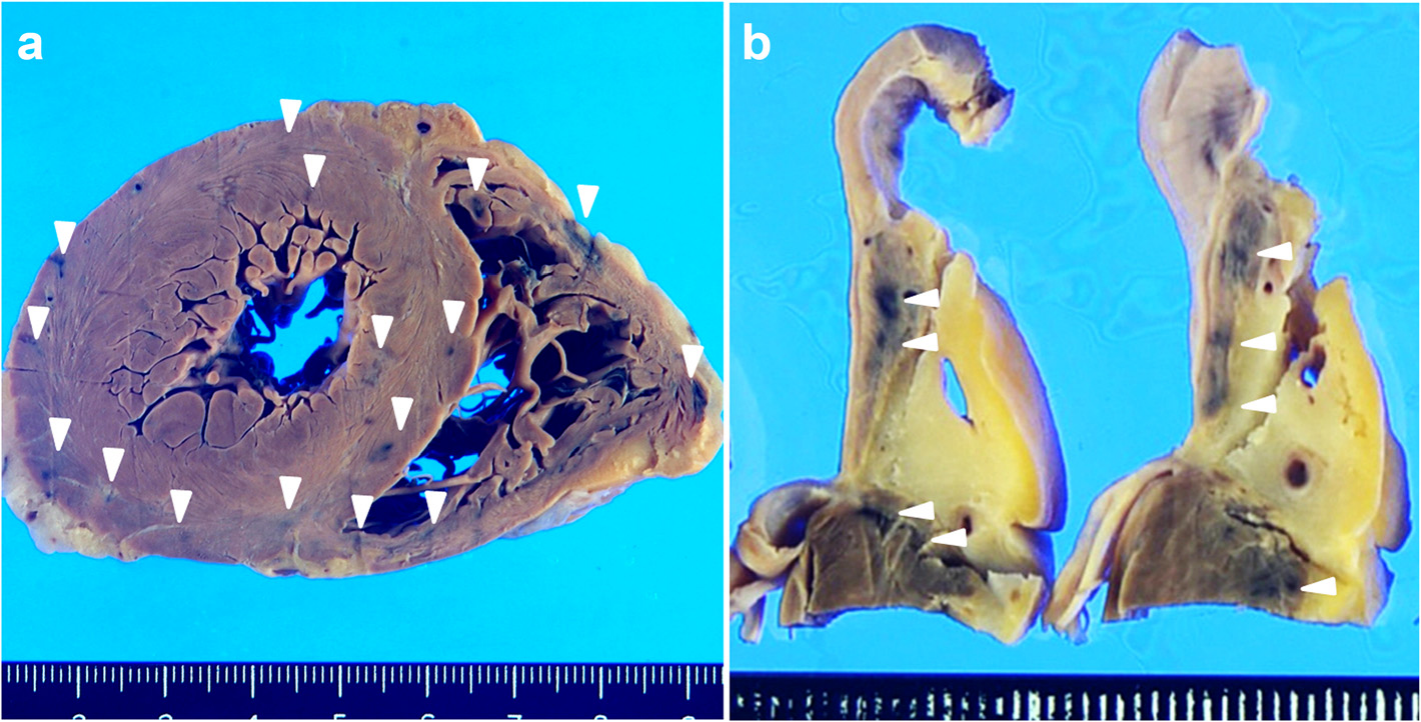
Macroscopic findings of the heart. **(a)** Concentric hypertrophy of the left ventricle is observed in the transverse section of the ventricle; many petechial hemorrhagic lesions (arrow head) are observed in both ventricle walls. **(b)** A sagittal section of the right atria and ventricles petechial hemorrhagic lesions (arrow head) are shown near the atrioventricular node.

**Figure 2:**
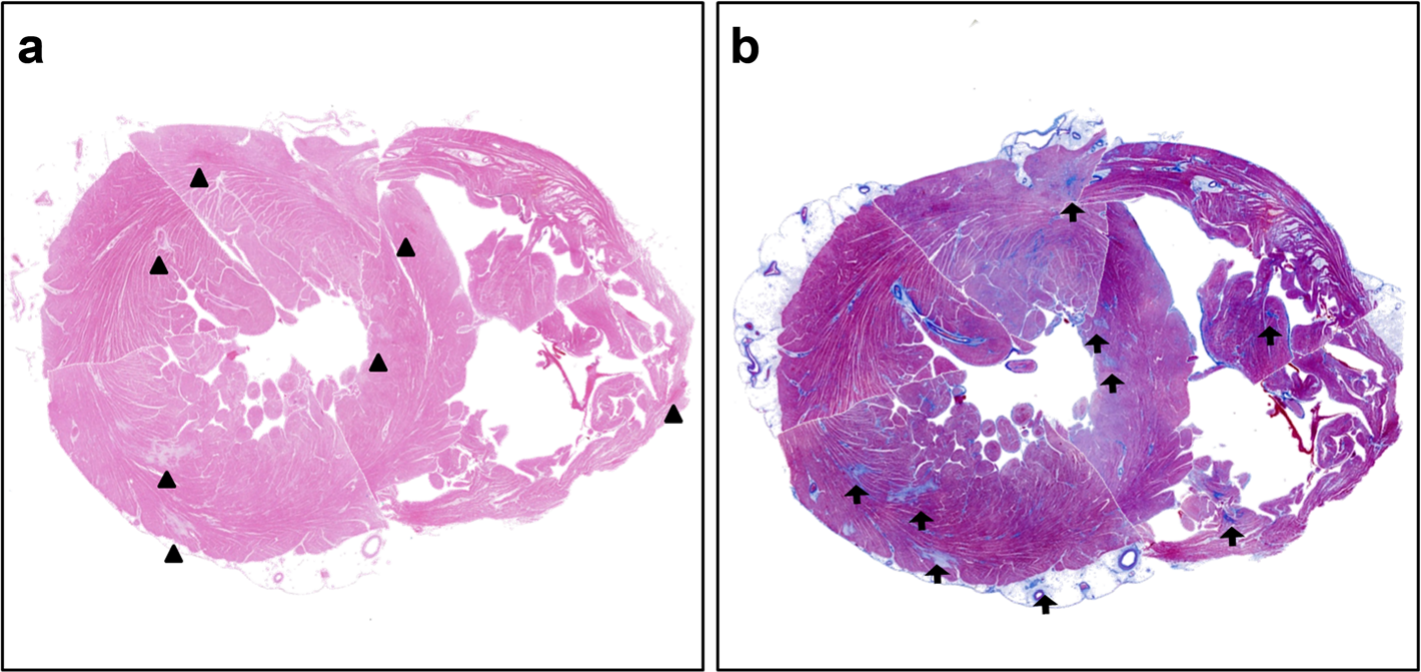
In scanning magnifications (1x) of the heart. **(a)** Many foci of necrosis with petechial hemorrhage ( arrow head) are observed. **(b)** many foci of necrosis with fibrosis (arrow) are visualized using Masson’s trichrome stain.

**Figure 3:**
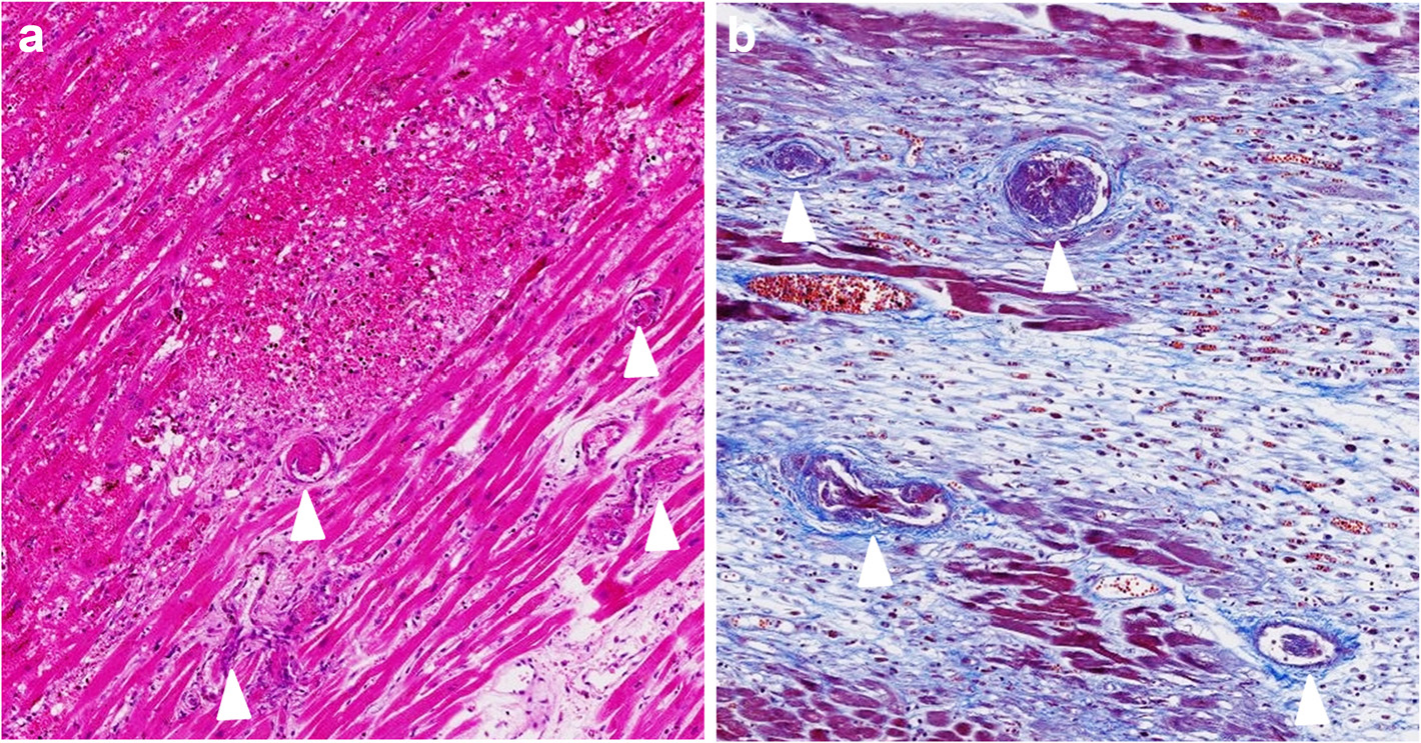
Medium power view (100x) of the heart. **(a)** The multiple thrombi (arrow head) of the capillary vessels or small arteries are observed in hemorrhage area. **(b)** Fibrotic foci around the multiple thrombi (arrow head) are detected by Masson’s trichrome stain.

**Figure 4:**
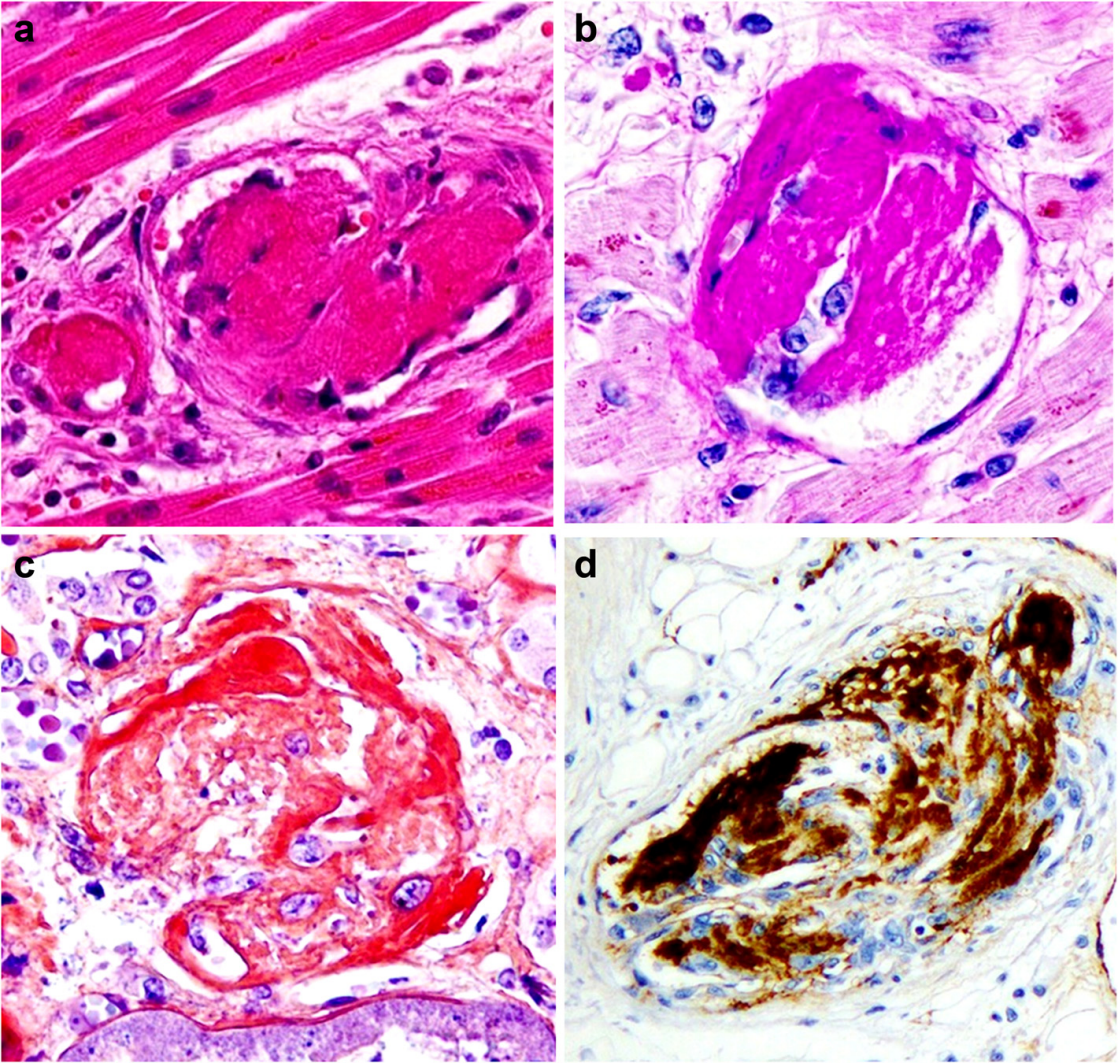
High power view (400x) of the heart. **(a)** An eosinophilic thrombus (arrow head) detected in the blood vessel, lined by enlarged endothelial cells. **(b)** The thrombus stains purple-red with the periodic acid Schiff stain. **(c)** The thrombus does not stain blue with phosphotungstic acid-hematoxylin stain. **(d)** The thrombus shows positive results for immunohistochemical staining for factor VIII.

The skin of the lower legs had disseminated petechial hemorrhages. Edema of the extremities was not observed. Macroscopically, the heart was enlarged (weighing 350 g) and the left ventricular wall was thickened (14 mm). Multiple petechial hemorrhages were diffusely distributed in the left and right ventricles and atria (Figure 1a, b). The left anterior coronary artery developed atheromatous lesions without significant stenosis. In the right atrium, near the atrioventricular node, a large number of hemorrhages were observed (Figure 1b). Only a small amount of pericardial effusion was present. Grossly-visible infarctions were not detected in other organs. According to the microscopic examination, numerous areas of hemorrhage (Figure 2a) and fibrosis (Figure 2b) were observed throughout the left and right ventricular and atrial myocardia, without any evidence of regional predominance. Several areas of spotty fibrotic foci (Figure 2b) were visualized using Masson’s trichrome stain. Hemorrhagic foci with infiltration of neutrophils, histiocytes, and lymphoplasmacytes and interstitial edema were observed (Figure 3a). In addition, fibrotic foci with fibroblastic proliferation, interstitial edema, and lymphoplasmacytic infiltration were observed (Figure 3b). Numerous hyaline microthrombi were observed in the intramural arterioles and capillaries, consisting of enlarged endothelial cells located adjacent to or within central hemorrhagic and fibrotic foci (Figure 4a). Microthrombi were positive for the periodic acid-Schiff stain (Figure 4b) and negative for phosphotungstic acid-hematoxylin stain (Figure 4c). According to the immunohistochemical study, microthrombi were strongly positive for factor VIII (Figure 4d). Therefore, hyaline thrombi predominantly consisted of platelets. In addition, hyaline intravascular microthrombi were also found in many other organs (i.e., liver, bilateral kidneys, spleen, pancreas, bilateral adrenal glands, gastrointestinal tract, prostate gland, skin of the abdominal wall, spinal cord, bone marrow of the lumbar vertebra, and paratracheal lymph node) even though infarction was not observed in any organs other than the heart. Microthrombi were also detected without fibrosis or congestion in the portal canals of the liver. Furthermore, microthrombi were noted in the capillaries of the renal cortical interstitial tissue, but not in the glomeruli. No remarkable changes were observed in the lungs. The brain could not be examined because his family’s consent was not obtained.

**Table 1:** Laboratory findings of the patient at presentation

Laboratory test	Data	Unit
WBC	7.1	×10^3^/μl
Hemoglobin	8.3	g/dl
Platelet	9	×10^3^/μl
Bilirubin (indirect)	2.3	mg/dl
AST	53	U/l
LDH	989	U/l
Creatinine	0.9	mg/dl
Prothrombin time	14.1	INR
APTT	27.5	s
CRP	3	mg/dl
Direct Coombs test	Negative	
Indirect Coombs test	Negative	
VWF cleaving protease infibitor	Positive	
Peripheral blood smear	Red blood cell fragmentation	

*WBC* white blood cells, *AST* asparate aminotransferase, *LDH* lactase dehydrogenase, *APTT* activated partial thromboplastin time, *CRP* C-reactive protein, *INR* international normalized ratio

It was suggested that the cause of sudden death might be arrhythmia by conduction system disorder and metachronous multiple cardiac infarction due to microthrombi, which resulted from idiopathic TTP.

## Discussion

TTP is a life-threatening disorder that is characterized by systemic platelet-VWF aggregation, organ ischemia, profound thrombocytopenia and fragmentation of erythrocytes [1]. In the present case, the patient exhibited 4 out of 5 classic symptoms of TTP: hemolytic anemia, thrombocytopenia, neurological abnormalities, and severe fever. He did not suffered from any diseases causing secondary TTP. Positive results were noted for the VWF cleaving protease inhibitor (anti-ADAMTS13 antibody), whereas the VWF cleaving protease (ADAMTS13) activity was markedly reduced. Upon the autopsy findings, platelet microthrombi were observed in many organs. Therefore, the patient was diagnosed as having idiopathic TTP.

This patient did not have symptoms of angina, myocardial infarction, or an elevation of cardiac enzymes. On the day before the patient’s death, atrial fibrillation was monitored on an ECG, but no ST segment elevation was noted. Contrary to the clinical features, severe cardiac infarction was observed histopathologically during the autopsy. According to the microscopic examination, the acute to subacute cardiac infarction manifested as hemorrhagic foci and chronic cardiac infarction manifested as fibrotic foci. Interestingly, it is typically that there were only a few occurrences of cardiovascular symptoms or events compared to the frequency of pathological myocardial infarction [7,8,12]. The reasons for this finding are currently limited. It is suggested that the ischemia results from small vessel occlusion by platelet thrombi and subsequent hypoxia. This is probably aggravated by the higher oxygen demand of the heart due to the associated anemia and peripheral tissue damage resulting from hypoxia. In fact, some previous reports have shown normal coronary arteries by angiograms in TTP patients with myocardial infarction [13,16]. It was probably difficult to assess the patient’s symptoms during the examinations (i.e., serological test and ECG), because the myocardial infarction in TTP was small in size and scattered. Myocardial damage can be a cause of death in patients with TTP, and cardiac dysfunction may persist in TTP survivors, although its potential impact on health and quality of life remains undetermined [14,19]. According to a published report, there was no significant difference in the classic cardiac risk factors between myocardial and non-myocardial infarction groups [12]. Considering this previous result, it is difficult to predict whether the myocardial infarction was caused by cardiac risk factors in TTP patients. Furthermore, the involvement of the cardiac conduction system is believed to be a cause of sudden death in patients with TTP, even though it is not often considered [9,14,20]. In our patient, the atrial fibrillation episode may have possibly led to the cardiac conduction system disorder. Although myocardial infarction was not clearly detected microscopically in the sinu-atrial (SA) node and the atrioventricular (AV) node, many infarctions were observed macro- and microscopically near the AV node. In this case, the sudden death in this patient with TTP was suggested to be caused by not only myocardial infarction, but possible cardiac conduction system disorder, according to the present autopsy findings. However, there has very rarely been a case report of the TTP patient who died suddenly, that the SA node and AV one were thoroughly examined by the subsequent autopsy, within our investigation.

It is important to ensure that the heart, including the SA node and AV one, is examined more closely in autopsy cases of patients with TTP who experience sudden death.

## Conclusion

The present patient was resistant to therapy and suddenly died within a short period. According to the pathological findings, platelet microthrombi were detected in multiple organs and many small infarctions were observed in the heart. Injury to other organs was limited, and therefore, it was possible that the sudden death was caused by myocardial infarction and an injury to the cardiac conduction system.

## Consent

Written informed consent was obtained from the patient for the publication of this report and any accompanying images.
